# BAP1 and UTX are dispensable for transcription-driven Polycomb erasure but essential for enhancer establishment

**DOI:** 10.1016/j.jbc.2026.113415

**Published:** 2026-08-07

**Authors:** Meihan Gong, Xuanyuan Li, Shaomei Chen, Xudong Wu

**Affiliations:** 1 Tianjin Medical University Cancer Institute & Hospital, National Clinical Research Center for Cancer, Tianjin’s Clinical Research Center for Cancer, The Province and Ministry Co-sponsored Collaborative Innovation Center for Medical Epigenetics, State Key Laboratory of Druggability Evaluation and Systematic Translational Medicine, Key Laboratory of Cancer Prevention and Therapy, Tianjin, China; 2 State Key Laboratory of Experimental Hematology, Key Laboratory of Immune Microenvironment and Disease (Ministry of Education), Tianjin Key Laboratory of Medical Epigenetics, Tianjin Medical University Cancer Institute and Hospital, Tianjin Medical University, Tianjin, China; 3 Department of Cell Biology, Tianjin Medical University, Tianjin, China

**Keywords:** eraser, bivalent chromatin, polycomb, enhancer establishment, H3K27me3, H2AK119ub

## Abstract

Polycomb group (PcG) proteins safeguard cell identity by maintaining target genes in a transcriptionally repressed state through depositing two histone marks, H2A lysine-119 ubiquitination (H2AK119ub1) and H3 lysine-27 trimethylation (H3K27me3). BAP1 and UTX are thought to erase these marks during transcription activation, but their exact roles remain unclear. Here, we observe a rapid loss of H2AK119ub1, followed by a reduction in H3K27me3 levels at promoters on transcription induction, coinciding with a swift dissociation of Polycomb repressive complexes (PRCs). With an acute degradation system, we show that neither BAP1 nor UTX is required for transcription activation or the dissolution of Polycomb group repressive marks, though BAP1 depletion leads to elevated steady-state H2AK119ub1 levels. Furthermore, while UTX regulates enhancer activation independently of its demethylase activity, BAP1 contributes to this process through a dual role: its scaffolding function supports MLL4 occupancy at nascent enhancers, whereas its catalytic activity restricts H2AK119ub1 accumulation that would otherwise impede enhancer maturation. Together, these findings establish that BAP1 and UTX are dispensable for transcription-driven erasure of Polycomb marks but are required for enhancer establishment, with BAP1 acting through coordinated scaffolding and catalytic mechanisms.

Histone post translational modifications are central to transcriptional regulation, allowing cells to dynamically adjust gene expression in response to developmental and environmental cues (1, 2). The enzymes that add (writers), remove (erasers), or recognize (readers) these marks work in concert to ensure dynamic and reversible control. Erasers are thought to be critical for resetting chromatin states (3, 4, 5). In line with this, eraser dysfunction is linked to cancer and neurological disorders. Understanding how erasers operate is therefore a key goal in chromatin biology.

Most studies of eraser function have relied on genetic knockouts. However, complete protein loss cannot distinguish whether observed phenotypes depend on catalytic activity or on nonenzymatic roles. Moreover, separating primary from secondary effects in such models is challenging.

It is widely assumed that for every repressive histone modification, dedicated erasers are required to reverse it upon gene activation. However, direct evidence for this assumption remains limited. The Polycomb group (PcG) system, with its two well-defined repressive marks and two candidate erasers, provides an ideal model to test this principle.

PcG proteins are conserved chromatin regulators that mediate transcriptional repression through two well-characterized histone modifications: H2A lysine-119 ubiquitination (H2AK119ub1) deposited by Polycomb repressive complex 1 (PRC1) (6, 7) and H3 lysine-27 trimethylation (H3K27me3) by PRC2 (8, 9, 10). These repressive marks cooperate to maintain gene silencing during development and cell fate decisions.

Conversely, two erasers have been proposed to counteract these repressive marks. KDM6A (UTX) and KDM6B (JMJD3) possesses a JmjC domain that selectively removes H3K27me3 (11, 12, 13, 14). They are found to play pivotal roles in various biological processes, particularly in embryonic differentiation, and is thought to relieve PcG-mediated gene silencing by erasing H3K27me3—for example, at bivalent promoters carrying both the repressive H3K27me3 mark and the activating H3K4me3 mark during differentiation induction (12, 13, 15, 16). Similarly, the Polycomb repressive deubiquitinase (PR-DUB) complex, in which BAP1 is the catalytic subunit, removes H2AK119ub1 (17, 18, 19). The ASXL subunit binds BAP1 *via* its DEUBAD domain and enhances its deubiquitinating activity (18, 20). Thus, bivalency resolution through the elimination of both H2AK119ub1 and H3K27me3 from target genes is a critical step during transcriptional induction in cellular differentiation. However, whether BAP1 and UTX are truly required as catalytic erasers during transcriptional induction, or whether they might play alternative roles independent of their enzymatic activity, remains unknown.

In this study, we find that transcription activation triggers a rapid, sequential loss of H2AK119ub1 followed by H3K27me3, which parallels the swift departure of PRC1 and PRC2 from target promoters. Using acute protein depletion, we demonstrate that neither BAP1 nor UTX is required for gene activation or for the removal of these repressive marks. Moreover, we uncover a dual role for BAP1 in enhancer establishment, acting through both catalytic and scaffolding mechanisms. Collectively, our results revise the current model: PcG mark resolution is driven by transcription, whereas BAP1 functions simultaneously as a scaffold for enhancer assembly and a gatekeeper of steady-state H2AK119ub1 levels.

## Results

### Rapid loss of H2AK119ub1 and subsequent deterioration of H3K27me3 during transcription induction

To study the dynamics of PcG marks during transcription activation, we established a rapid induction model by treating wild-type (WT) embryonic stem cells (ESCs) with retinoic acid (RA) for 0, 3, and 6 h (h). Genes induced after 6 h of RA stimulation were overlapped with known H3K27me3 targets from our previous study and defined as “RA-inducible PcG targets” (Fig. S1*A*) (21). Representative target genes such as *Hoxa1*, *Hoxb1*, and *Cyp26a1* show a time-dependent increase in expression, as confirmed by independent Reverse Transcription quantitative Polymerase Chain Reaction (RT-qPCR) analysis (Fig. S1, *B* and *C*). *Gata4* serves as a negative control.

**Figure 1:**
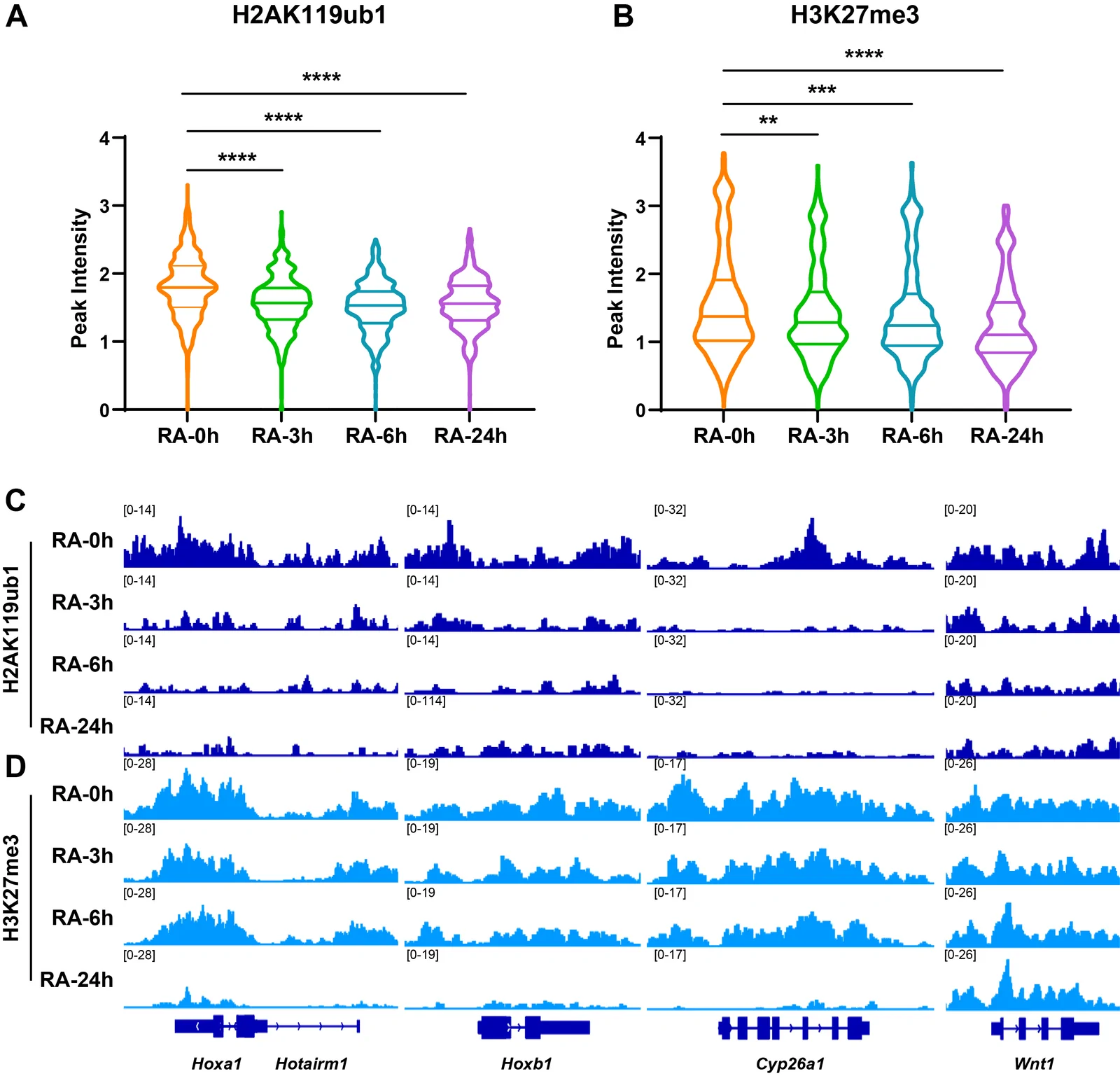
**Induced transcription drives rapid loss of H2AK119ub1 and subsequent depletion of H3K27me3.***A*-*B*. violin plots showing ChIP-seq signal intensity (log_2_ (RPKM + 1)) for H2AK119ub1 (*A*) or H3K27me3 (*B*) at RA-inducible Polycomb targets in WT ESCs at 0, 3, 6, or 24 h of RA treatment. The *central line* indicates the median; the *box* spans the 25th to 75th percentiles. Statistical significance was determined by Wilcoxon rank-sum test: ∗*p* < 0.05, ∗∗*p* < 0.01, ∗∗∗*p* < 0.001, ∗∗∗∗*p* < 0.0001. *C*-*D*. genome browser views of H2AK119ub1 (*dark blue*) or H3K27me3 (*light blue*) enrichment at representative loci in WT ESCs following RA treatment for 0, 3, 6, and 24 h *Wnt1* is served as a negative control. ChIP-seq, chromatin immunoprecipitation sequencing; ESC, embryonic stem cell; RA, retinoic acid.

We next performed chromatin immunoprecipitation sequencing (ChIP-seq) for H2AK119ub1 and H3K27me3 on WT ESCs treated with RA for 0, 3, 6, and 24 h. At RA-inducible PcG target promoters, enrichment of both repressive marks gradually decreases upon RA stimulation. Notably, H3K27me3 levels at 3 h show a statistically significant reduction compared to 0 h (*p* < 0.01). However, the magnitude of this reduction was modest, whereas H2AK119ub1 levels at 3 h dropped to near-background levels (*p* < 0.0001). Importantly, H3K27me3 did not reach a comparable level of depletion until 24 h of RA stimulation. (Fig. 1, *A*–*D*). These results indicate that H2AK119ub1 turns over faster than H3K27me3 in response to an external signaling cue.

### Loss of BAP1 or UTX does not affect transcriptional induction of PcG targets

**Figure 2:**
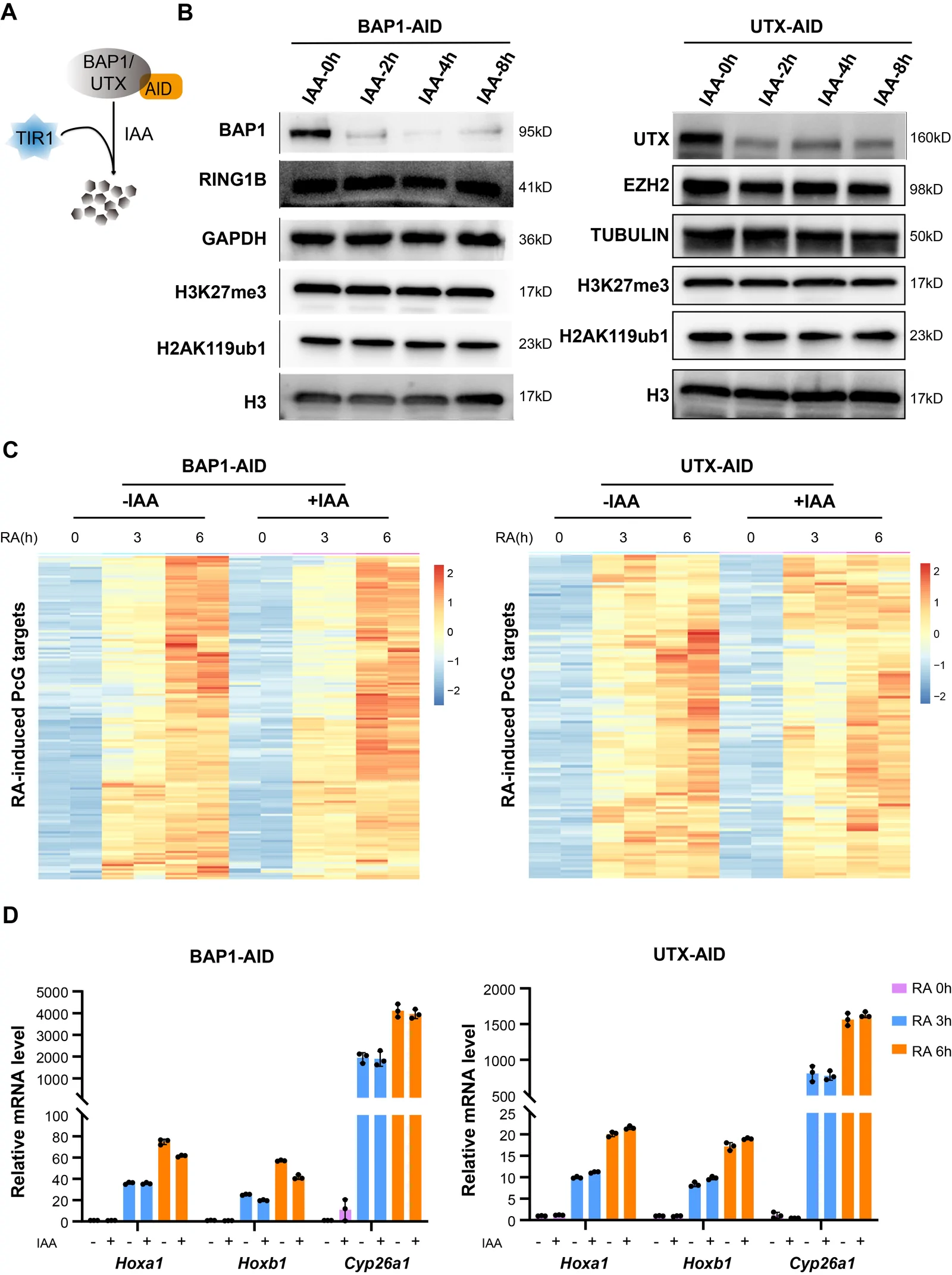
**BAP1 or UTX loss does not significantly affect the transcriptional induction of PRC target genes in response to retinoic acid.***A*, schematic of the AID system for BAP1 and UTX. Addition of 100 μg/ml auxin IAA to cells expressing the TIR1 ubiquitin ligase induces rapid proteasomal degradation of AID-tagged BAP1 and UTX. *B*, WB assays to detect the levels of designated proteins in BAP-AID or UTX-AID ESCs treated with IAA for 0, 2, 4, and 8 h. *C*, expression dynamics of RA-inducible Polycomb target genes in BAP1-AID or UTX-AID ESCs. Cells were pretreated with or without IAA for 2 h, followed by differentiation induction with 2 μM RA for 3 or 6 h. *D*, RT-qPCR analysis of *Hoxa1*, *Hoxb1*, and *Cyp26a1* mRNA levels at 3 h and 6 h of RA treatment in BAP1-AID or UTX-AID ESCs following IAA-mediated protein depletion. AID, auxin-inducible degradation; ESC, embryonic stem cell; IAA, indole-3-acetic acid; PRC, Polycomb repressive complex; RA, retinoic acid; RT-qPCR, Reverse Transcription quantitative Polymerase Chain Reaction; WB, western blot.

Next, we asked whether BAP1 and UTX are required for the removal of these marks upon transcriptional induction. To address this, we used an auxin-inducible degradation (AID) system to achieve rapid protein loss (22). We generated BAP1-AID and UTX-AID ESCs by introducing an HA-AID tag at the C terminus of BAP1 or the N terminus of UTX using CRISPR-Cas9 (Figs. 2*A* and S2*A*). Correct integration was confirmed by genotyping PCR (a ∼100 bp size shift) (Fig. S2, *A* and *B*) and sequencing. TIR1, the E3 ligase adaptor required for auxin-induced degradation (22), was stably overexpressed in the validated clones (Fig. 2*A*). Treatment with indole-3-acetic acid (IAA) for 2 h led to efficient degradation of BAP1 or UTX, without affecting global levels of H2AK119ub1, H3K27me3, RING1B (PRC1 core component), or EZH2 (PRC2 core component) (Fig. 2*B*).

To test the impact of BAP1 or UTX loss on RA-inducible PcG targets, cells were pretreated with IAA for 2 h before RA stimulation, and RNA-seq analysis was performed at 0, 3, and 6 h post stimulation. As illustrated as heatmap, degradation of BAP1 or UTX does not significantly alter the activation of RA-inducible PcG targets compared to control cells (Fig. 2*C*). RT-qPCR analysis on representative genes confirmed similar expression trends (Fig. 2*D*). Thus, neither BAP1 nor UTX is strictly required for stimulation-induced transcription of PcG targets.

### RA-induced bivalency resolution is driven by rapid, transcription-dependent PRC1/2 dissociation, rather than BAP1 or UTX loss

**Figure 3:**
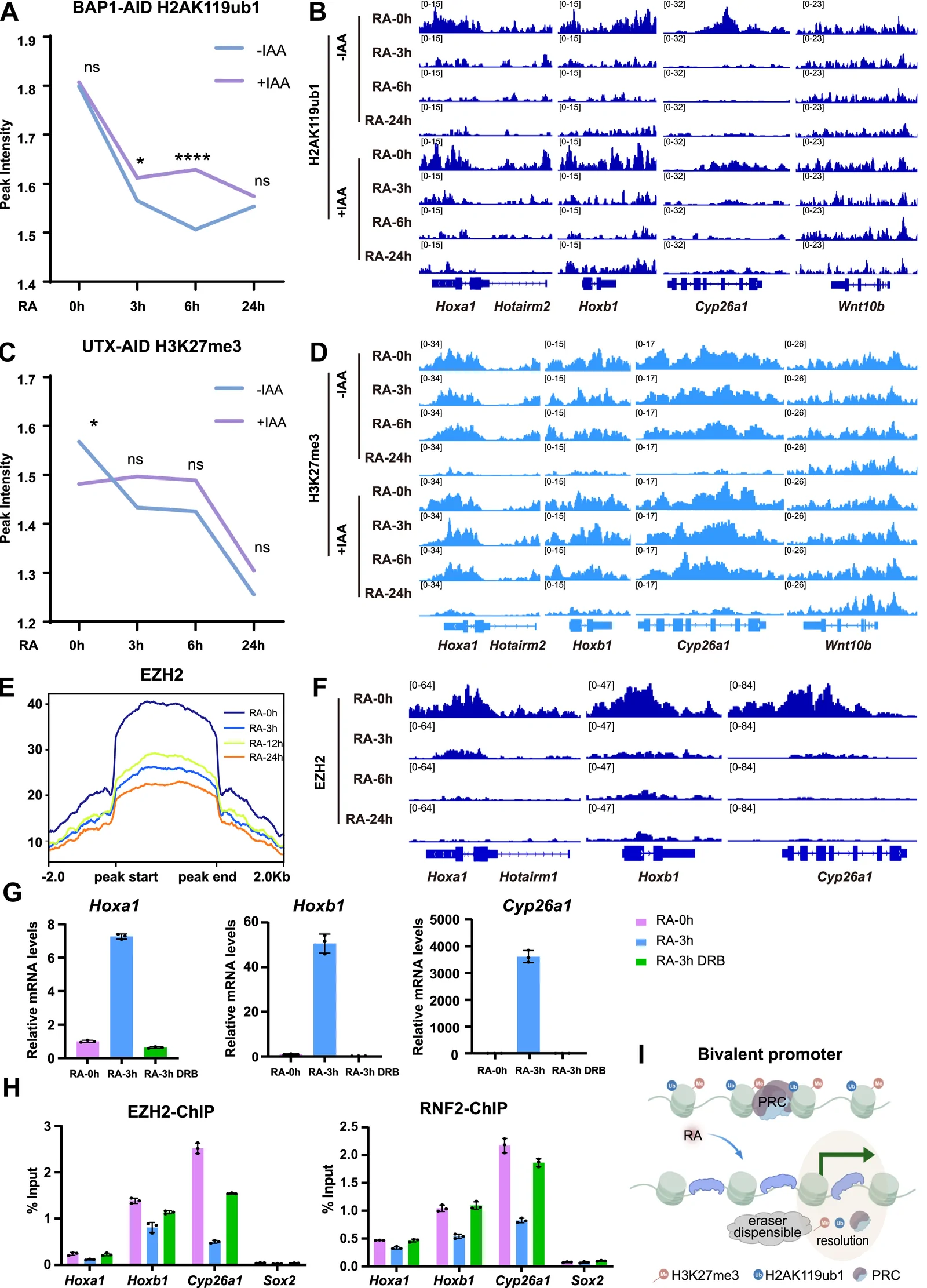
**RA-induced bivalency resolution is driven by rapid, transcription-dependent PRC dissociation, rather than BAP1 or UTX loss.***A*, *C*. Log_2_ (RPKM+1) values of H2AK119ub1 or H3K27me3 ChIP-seq signals are shown at RA-inducible PcG targets across a time course of retinoic acid (RA) stimulation (0, 3, 6, 24 h), with or without IAA-mediated degradation of BAP1 (*A*) or UTX (*C*). The *blue line* represents the control condition (–IAA); the *purple line* indicates the condition with IAA treatment (+IAA). Statistical significance was determined by two-sided unpaired *t* test: ∗*p* < 0.05, ∗∗*p* < 0.01, ∗∗∗*p* < 0.001, ∗∗∗∗*p* < 0.0001. *B* and *D*. representative genome browser tracks showing H2AK119ub1 or H3K27me3 enrichment at exemplary target genes in BAP1-AID (*B*) or UTX-AID (*D*) ESCs. *Wnt10b* serves as a negative control. *E*, Line charts showing average EZH2 signals at RA-inducible PcG targets in WT ESCs. The *x* axis represents the distance to peak start and end. *F*, representative genome browser tracks showing dynamic loss of EZH2 enrichment at *Hoxa1*, *Hoxb1*, and *Cyp26a1* in WT ESCs. *G*, RT-qPCR analysis of the dynamic expression of indicated genes (*Hoxa1, Hoxb1,* and *Cyp26a1*) following 2 μM RA treatment for 3 h or 100 μM DRB treatment for 3 h concurrently in WT ESCs. *H*, ChIP-qPCR analysis of EZH2 and RNF2 occupancy at the indicated gene promoters under the same treatment conditions as in (*G*). *I*, schematic model: Transcription activation of bivalent genes drives the dissociation of PRCs from target promoters, resulting in the resolution of associated repressive histone marks independent of erasers. AID, auxin-inducible degradation; ChIP, chromatin immunoprecipitation; DRB, 5,6-dichloro-1-beta-D-ribofuranosylbenzimidazole riboside; ESC, embryonic stem cell; IAA, indole-3-acetic acid; PRC, Polycomb repressive complex; RA, retinoic acid; RT-qPCR, Reverse Transcription quantitative Polymerase Chain Reaction.

We next asked whether loss of BAP1 or UTX affects the temporal removal of H2AK119ub1 and H3K27me3 from promoters during transcription activation. BAP1-AID and UTX-AID cells were treated with or without IAA, followed by RA for 0, 3, 6, or 24 h. ChIP-seq analysis for H2AK119ub1 showed that BAP1 degradation did not prevent the reduction of this mark at RA-inducible PcG target promoters; the kinetics were similar to those in the presence of BAP1 (Fig. 3, *A* and *B*). Chromatin immunoprecipitation qPCR (ChIP-qPCR) analysis confirmed these results (Fig. S3*A*). Hence, even without BAP1, H2AK119ub1 is efficiently removed upon signaling, coincident with normal transcriptional activation. Given that BAP1 primarily restricts H2AK119ub1 at a genome-wide level (23, 24, 25, 26), the slight but significant increase in H2AK119ub1 signal upon BAP1 depletion (Fig. 3, *A* and *B*) is likely attributable to incomplete removal of steady-state H2AK119ub1, rather than to effects on mark turnover during activation.

Similarly, in UTX-AID cells, degradation of UTX does not impair the loss of H3K27me3 from RA-inducible PcG target promoters after RA treatment (Fig. 3*, C* and *D*). Chromatin immunoprecipitation qPCR analysis on *Hoxa1*, *Hoxb1*, and *Cyp26a1* promoters validated this observation, with *Sox2* as a negative control (Fig. S3*B*). Notably, the lack of effect is not due to compensation by its homologous proteins, as UTY is not expressed in E14 cells whereas JMJD3 expression remains unchanged after UTX degradation (Fig. S3*C*). Together, these data indicate that the catalytic activities of BAP1 and UTX are dispensable for removing Polycomb-mediated modifications on transcription induction.

We and others have previously shown that PRCs sense the transcriptional state of chromatin (27, 28, 29). We therefore hypothesized that the loss of repressive marks might simply reflect PRC dissociation. Indeed, EZH2 ChIP-seq analysis revealed that, unlike H3K27me3, EZH2 enrichment at RA-inducible PcG target promoters is already markedly reduced at 3 h of RA stimulation (Fig. 3*, E* and *F*). Chromatin immunoprecipitation qPCR analysis for both EZH2 and RING1B confirmed this rapid dissociation (Fig. S3*D*). Thus, gene activation is accompanied by transcription-driven release of PRC1 and PRC2 from chromatin.

To further test whether PRC dissociation depends on active transcription, we treated cells with RA together with 5,6-dichloro-1-beta-D-ribofuranosylbenzimidazole riboside, an inhibitor of RNA polymerase II Ser2 phosphorylation that blocks transcriptional elongation (30). 5,6-dichloro-1-beta-D-ribofuranosylbenzimidazole riboside effectively suppresses RA-induced gene activation (Fig. 3*G*). Under these conditions, the RA-mediated dissociation of EZH2 and RING1B from target promoters is largely prevented (Fig. 3*H*). These results confirm that PRC dissociation from target promoters is transcription-dependent.

Notably, H3K27me3 is also present at poised enhancers. We asked whether it is erased by UTX or JMJD3 during enhancer activation. Using a previously generated inducible CRISPR activation system (iVPR mESCs) (31), we activated poised enhancers of two representative genes (*T*, *NKX2.5*) with doxycycline (DOX) treatment (Fig. S4*A*). Pretreatment with the KDM6 inhibitor GSK-J4 for 24 h does not affect either global H3K27me3 levels, or doxycycline-induced gene activation, H3K27me3 depletion, H3K4me1 establishment at the enhancer loci (Fig. S4*, B*–*E*). Therefore, H3K27 demethylases are also dispensable for resolving repressive marks at poised enhancers under these experimental conditions. Collectively, these findings indicate that transcription activation drives rapid PRC dissociation, leading to eraser-independent resolution of PcG-mediated modifications (Fig. 3*I*).

### BAP1 facilitates enhancer establishment through both catalytic and scaffolding functions

UTX is a component of the COMPASS complex and shows extensive genomic overlap with MLL4 and p300. Notably, UTX knockout reduces MLL4 and p300 enrichment at active enhancers and regulates enhancer activation independent of its demethylase activity (32, 33, 34). In line with the critical roles of MLL3/MLL4 at enhancers (35), these observations suggest that UTX facilitates enhancer establishment by recruiting COMPASS components. Recent studies have also demonstrated that the MBH domain of ASXL proteins directly interact with MLL3/4, mediating their cooccupancy at enhancer regions (36, 37, 38). These observations suggest that BAP1 may similarly exert additional, noncatalytic functions in enhancer regulation.

**Figure 4:**
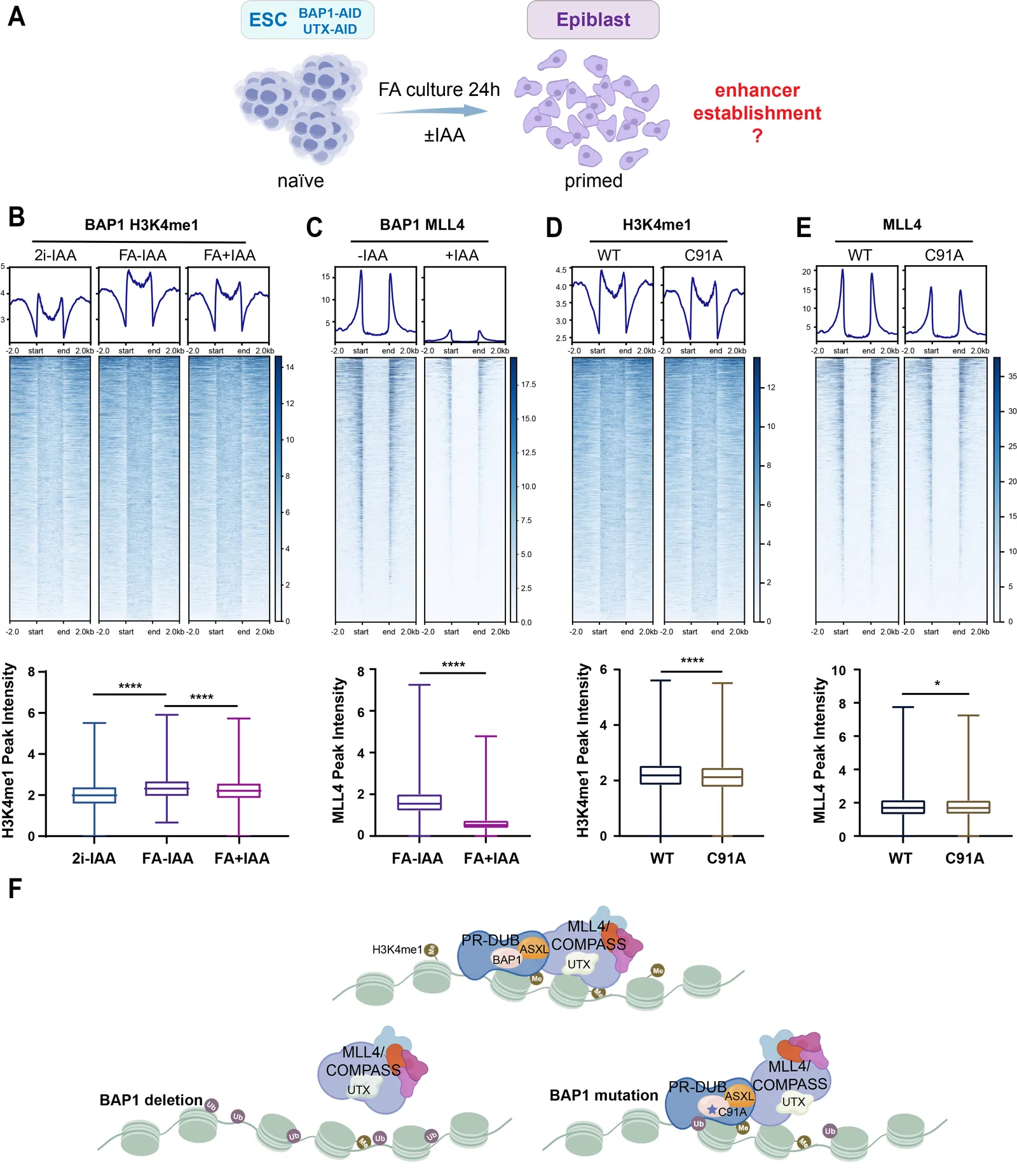
**BAP1 facilitates enhancer establishment through both catalytic and scaffolding functions.***A*, schematic overview of the *in vitro* differentiation paradigm from naïve ESCs (maintained in 2i/LIF) to a primed epiblast-like state (induced by 24-h FGF/Activin A [FA] treatment). *B*, heatmaps depicting H3K4me1 ChIP-seq signal dynamics at H3K4me1-gain peak regions during 24 h FA culture condition compared to 2i condition in control (−IAA) *versus* BAP1-depleted (+IAA) AID ESCs. The corresponding statistical results are shown in the lower panel. Quantitative comparison of H3K4me1 signal intensity (log_2_ (RPKM+1)) at H3K4me1-gain regions Data are presented as boxplots; error bars represent standard deviation (SD). Statistical significance was determined by Wilcoxon rank-sum test: ∗*p* < 0.05, ∗∗*p* < 0.01, ∗∗∗*p* < 0.001, ∗∗∗∗*p* < 0.0001. This statistical method was applied to all subsequent panel. *C*, heatmaps showing MLL4 CUT&Tag signals at H3K4me1-gain regions during 24 h FA-induced differentiation in BAP1-AID ESCs with IAA treatment. Statistical analysis for the above data is provided in the lower panel. *D*-*E*. heatmaps showing H3K4me1 ChIP-seq or MLL4 CUT&Tag signals in reintroduced WT and C91A BAP1-AID ESCs with IAA treatment during 24 h FA differentiation. The lower panel presents associated statistical analysis. *F*, schematic model: BAP1 functions through dual mechanisms in enhancer establishment. Its deubiquitinase activity restricts steady-state H2AK119ub1 genome-wide, preventing aberrant H2AK119ub1 retention that would otherwise antagonize COMPASS activity and impair enhancer maturation. More importantly, its scaffolding function directly facilitates MLL3/4 recruitment, thereby promoting COMPASS-mediated enhancer assembly. Thus, BAP1 licenses proper enhancer formation through coordinated catalytic and structural roles. AID, auxin-inducible degradation; ChIP, chromatin immunoprecipitation; ESC, embryonic stem cell; IAA, indole-3-acetic acid.

Given that RA-responsive ectodermal enhancers are already preestablished in ESCs (39, 40), this system precludes the study of *de novo* enhancer formation. To test whether BAP1 is similar to UTX and functionally required for enhancer establishment during cell fate transition, we subjected the AID ESCs to a Fgf2 and activin (FA)-induced differentiation protocol (41). Cells were cultured under FA conditions for 24 h to monitor enhancer dynamics, with or without prior IAA treatment for 24 h to degrade the target proteins (Figs. 4*A* and S5). We performed H3K4me1 ChIP-seq and defined “H3K4me1-gain regions” as genomic loci with increased H3K4me1 signal upon FA induction compared to baseline 2i conditions.

Consistent with previous findings, acute UTX loss prevents FA-driven MLL4 deposition and enhancer formation (Fig. S6*, A* and *B*). These defects are specifically rescued by WT-UTX, but not by its catalytically inactive mutant (dMT: H1146A/E1148A) (Fig. S6*, C* and *D*), confirming that UTX regulates enhancer establishment in a demethylase-independent manner. Similarly, BAP1 degradation significantly impairs the normal upregulation of H3K4me1 levels at these regions (Fig. 4*B*), indicating that BAP1 is essential for proper enhancer establishment. Consistent with its role in MLL3/4 recruitment at enhancers, MLL4 enrichment is markedly reduced at the same H3K4me1-gain regions upon BAP1 loss (Fig. 4*C*).

Although BAP1 is dispensable for active H2AK119ub1 removal during transcriptional activation, its depletion leads to a modest but significant elevation of H2AK119ub1 levels at promoters (Fig. 3*, A* and *B*), suggesting a role in restricting steady-state ubiquitination. To determine whether BAP1's enzymatic activity is required for enhancer establishment, we performed rescue experiments in BAP1-AID ESCs by reintroducing either WT or catalytically dead mutants–C91A. As expected, H2AK119ub1 accumulates globally in the C91A group (Fig. S5). After 24 h of FA-induced differentiation, we reassessed enhancer states by ChIP-seq analysis. Remarkably, the C91A construct only partially restores H3K4me1 gain and MLL4 occupancy at these enhancer regions upon IAA treatment, with rescue being significantly weaker than that achieved by WT BAP1 (Fig. 4, *B*–*E*). These findings strongly support a model in which BAP1 facilitates enhancer formation: the scaffold function of BAP1 predominates in enhancer establishment, whereas its catalytic activity plays a supportive role by preventing aberrant H2AK119ub1 accumulation that would otherwise impede enhancer maturation (Fig. 4*F*). Together, these data indicate that UTX acts primarily as a bridging factor that recruits the COMPASS complex to establish enhancers during cell fate transition, whereas BAP1 plays both enzymatic and scaffolding roles in enhancer establishment.

## Discussion

The classical model holds that PRC1 and PRC2 maintain gene silencing by catalyzing H2AK119ub1 and H3K27me3, respectively, whereas BAP1 and UTX act as dedicated erasers to remove these modifications upon activation (12, 13, 15, 19, 42, 43). However, our findings indicate that the sequential resolution of H2AK119ub1 and H3K27me3 is not necessary for the activation of PcG-target genes. Instead, their resolution is transcription-driven.

Our time-resolved analyses revealed a notable kinetic difference: H2AK119ub1 loss (within 3 h) but a much slower decline of H3K27me3 (by 24 h). Although we cannot exclude the possibility of the rapid action of USP family deubiquitinases (44, 45, 46, 47), this difference likely reflects distinct turnover rates of their underlying histones. H2AK119ub1 is deposited on canonical H2A or H2A.Z, which turns over relatively quickly (48), whereas H3K27me3 resides on H3.1 and H3.3, which have slower exchange rates (49, 50, 51). Thus, the distinct kinetics we observe are most simply explained by intrinsic histone turnover rather than by differential eraser activity. In this context, BAP1's primary function appears to be restricting steady-state H2AK119ub1 levels genome-wide, rather than driving acute mark removal upon activation.

Consistent with our findings, KDM6 activity has been demonstrated demethylase-activity independent roles in embryonic development and lineage differentiation in different species (52, 53, 54, 55, 56). Extending this principle, we uncover an unexpected, enzymatic-activity-independent and dependent role for BAP1 in establishing enhancers during cell fate transitions. It raises the intriguing possibility that other histone modification erasers similarly possess noncatalytic scaffolding functions. Given that many erasers contain protein-interaction domains, such moonlighting functions could be widespread.

For H3K27 demethylation, our study focuses primarily on UTX rather than JMJD3 based on the following considerations. First, JMJD3 is generally expressed at low levels in undifferentiated ESCs and is strongly induced only upon differentiation or inflammatory stimulation (12, 14). Second, genetic evidence reveals distinct phenotypes: UTX-null female mice die at E11.5 from severe mesodermal defects (52), whereas JMJD3-null embryos survive to birth with only postnatal respiratory failure (57). These observations suggest that UTX plays a broader and more fundamental role in early cell fate decisions, making it a suitable model to examine its significance in bivalency resolution. Third, UTX is a stable component of the COMPASS complex and has been shown to possess noncatalytic scaffolding functions in enhancer regulation (32, 33, 34). Whether JMJD3 also contributes to transcription activation or enhancer establishment in contexts where UTX is limiting remains an open question.

Several limitations should be acknowledged. First, although our data suggest a scaffolding role for BAP1 in enhancer establishment, we lack direct evidences such as physical interaction data or chromatin recruitment assays to confirm this mechanism. Second, while the partial catalytic requirement may reflect BAP1's role in restricting steady-state H2AK119ub1, we cannot conclude that this occurs specifically at enhancer loci due to rather low H2AK119ub1 enrichment (data not shown). Future studies using targeted protein interaction mapping and locus-specific recruitment will be needed to fully dissect these functions.

While we clearly demonstrate eraser-independent roles during rapid transcription induction, we do not argue that the catalytic activities of BAP1 and UTX are entirely dispensable in all biological contexts. For example, the two PcG repressive marks can become decoupled at late developmental stages. PR-DUB has been shown to remove H2AK118ub1 mark from PcG-target sites (26, 58), though the significance remains not completely understood. Similarly, the demethylase activity of KDM6 has been shown to be critical for neurogenesis, cellular senescence, metabolism, tissue regeneration and repair, and so on (59, 60, 61, 62). Careful profiling of dynamic chromatin changes and well-designed experimental settings will be required to dissect the regulatory mechanisms underlying these eraser-activity-dependent roles.

In summary, our findings overturn the long-held assumption that BAP1 and UTX are essential for removing repressive marks during transcriptional induction. Instead, they reveal a transcription-driven mechanism of PRC eviction and assign BAP1 a dual function in enhancer establishment. These insights refine our understanding of Polycomb biology and have therapeutic implications, as the scaffolding activities of BAP1 may be the relevant drivers in cancers where these proteins are mutated or overexpressed.

## Experimental procedures

### Cell culture and differentiation

Mouse ESCs were cultured in serum/leukemia inhibitory factor medium seeded on 0.1% gelatin coated plates as previously described (41). Cells were cultured at 37 °C with 5% CO2. To generate BAP1-AID or UTX-AID ESCs, a single guide RNA (sgRNA) and a donor plasmid containing hemagglutinin-AID tag were transfected into ES cells dish using Lipofectamine 3000 transfection reagent. Specific sgRNA was designed by at the website of http://crispor.tefor.net/. sgRNA oligos were annealed and then cloned into vector according to Zhang lab’ protocol. The construction of donor-containing plasmids was achieved through homologous recombination. Then positive monoclonal DNA were extracted and identified by PCR following sequencing analysis.

To induce target protein degradation, ethanol-dissolved IAA was mixed with cell medium to a final concentration of 100 μg/ml. For RA induced differentiation, the final concentration of 2 μM is used without leukemia inhibitory factor. For the switch of naïve to primed state, medium was converted to serum-free medium with N2 and B27 supplements, Fgf2 (12 ng/ml) and activin A (20 ng/ml).

### RNA extraction and RT-qPCR

Total RNA was isolated with TRIzol (GenStar, P118-05). Then 1 μg RNA was reversed transcription (TransGen Biotech, AU341) and obtained complementary DNA were performed quantitative real-time PCR using 2× ChamQ Universal SYBR qPCR Master Mix (Q711, Vazyme) on LightCycler 480 system of Roche. The primer sequence was list in Table S1.

### RNA sequencing (RNA-seq) and data analysis

Total RNA was extracted using TRIzol reagent in strict accordance with the manufacturer’s instructions. The sequencing data were aligned to the mm39 reference genome using HISAT2 (version 2.2.1), and Samtools (version 1.19) was utilized to convert SAM files to BAM files. deepTools (version 3.5.4) was employed to generate bigwig files, and visualization of the data was performed using the Integrative Genomics Viewer (IGV). Gene expression counts were calculated by StringTie (version 2.2.1), and differential expression analysis was conducted with the R package DESeq2 (version 1.34.0). Genes were considered significantly differentially expressed if they satisfied the criteria of padj < 0.05 and |log2FoldChange| > 1.

### Chromatin immunoprecipitation (ChIP)-qPCR

ChIP assay was performed as described previously (41, 63). Briefly, ESCs were crosslinked with 1% formaldehyde for 10 min (min) at room temperature. Then glycine was added to stop fixation with 0.125 M final concentration and incubated for 5 min. Next, cells were washed twice with PBS, and the cell pellets were resuspended and lyzed with SDS buffer (100 mM NaCl, 50 mM Tris–HCl, pH = 8.1, 5 mM EDTA, 1% SDS, and 1× protease inhibitor cocktail (PIC)). After collected by centrifugation at 2000 rpm for 5 min, chromatin was resuspended in suitable volume IP buffer (SDS buffer: Triton buffer (100 mM NaCl, 100 mM Tris–HCl, pH = 8.6, 5 mM EDTA, 5% Triton X-100, 1× protease inhibitor cocktail) = 2:1). Then chromatin fragmentation was performed using BioRuptor sonicator (Diagenode) to an average 200 to 500 bp; appropriate amounts were used for the following experiments. Primary antibodies were added to chromatin samples at 4 °C overnight. The next day, 30 ul of protein A/G magnetic beads were added for IP at 4 °C for 2 h rotating. Then the beads were washed once with wash buffer Ⅰ (low salt buffer: 0.1% SDS, 1% Triton X-100, 150 mM NaCl, 2 mM EDTA, 20 mM Tris–HCl, pH = 8.0) and twice with wash buffer Ⅱ ((high salt buffer: 0.1% SDS, 1% Triton X-100, 500 mM NaCl, 2 mM EDTA, 20 mM Tris–HCl, pH = 8.0). ChIP DNA was purified after de-crosslinking at 65 °C overnight and followed by quantitative PCR (qPCR) analysis or library preparation for high-throughput sequencing. Primer sequences for qPCR analysis are listed in Table S1. Data were obtained and calculated. The primary antibodies used in this study are listed in Table S2.

### CUT&Tag

The CUT&Tag assay was performed using the Hyperactive Universal CUT&Tag Assay Kit (Vazyme, TD904) according to the manufacturer’s instructions, with minor modifications. Briefly, 100,000 cells were harvested by centrifugation, washed once with 1× Wash Buffer, and resuspended in 100 μl of Wash Buffer. 10 μl of preactivated Concanavalin A magnetic beads were added to the cell suspension and incubated for 10 min at room temperature with gentle rotation to allow cell-bead conjugation. The supernatant was removed by placing the tubes on a magnetic rack, and the bead-bound cells were resuspended in 50 μl antibody binding buffer. Primary antibody was added, and the mixture was incubated overnight at 4 °C with rotation. On the following day, the supernatant was removed after magnetic separation, and the bead-cell complexes were washed briefly before incubation with 50 μl of DIG Wash Buffer containing secondary antibody for 1 h at room temperature. The beads were subsequently washed three times with 200 μl DIG Wash Buffer, then resuspended in 100 μl Dig-300 Buffer containing pA/G-Tn5 Pro transposase (0.04 μM) and incubated for 1 h at room temperature. After three additional washes with 200 μl Dig-300 Buffer, the beads were resuspended in 40 μl Dig-300 Buffer, and 10 μl of 5× TTBL Buffer was added to initiate tagmentation at 37 °C for 1 h. Following brief centrifugation, 2 μl of 10% SDS and an appropriate volume of DNA Spike-in control (adjusted based on the expected abundance of the target protein and cell number) were added to the reaction, mixed by gentle inversion, and incubated at 55 °C for 10 min to inactivate the Tn5 transposase and release tagmented DNA. The mixture was briefly vortexed or inverted 2 to 3 times during incubation to ensure homogeneity. The DNA was then purified using DNA Extract Beads Pro according to the kit protocol. Purified DNA fragments were amplified by PCR to generate sequencing libraries, which were subsequently sequenced on an Illumina PE150 platform. The primary antibodies used in this study are listed in Table S2.

### ChIP-seq data analysis

Raw data underwent quality evaluation with Fastqc (v0.12.1) and Multiqc (v1.19). ChIP-seq reads were first processed using Trim-galore (v0.6.10) to trim adaptor and low-quality reads. Duplicates removed with Sambamba (v1.0). Trimmed reads were aligned to the mouse reference genome mm39. Peaks were called using MACS2 (v2.2.9.1) and broad peak calling for H2AK119ub1. Signal tracks were normalized using counts per million and track (bigwig) files were generated using bamCoverage from deepTools (v3.5.4). These normalized data were further used for plot heatmaps or boxplots.

### CUT&Tag data analysis

Raw data underwent quality evaluation with Fastqc (v0.12.1) and Multiqc (v1.19). Adapters were removed with Trim-galore (v0.6.10). Clean reads were aligned to the mm39 reference genome and λdna/S2 by Bowtie2(v2.5.3) with --end-to-end --very-sensitive --no-mixed --no-discordant --no-unal -I 10 -X 700. Duplicates removed with Sambamba (v1.0), then bigwig files were generated with deepTools (v3.5.4), scale-factor for normalized was calculated based on spike-in alignment reads. Peak calling was conducted with Macs2 (v2.2.9.1), Peaks were annotated with R packages ChIPseeker (v1.30.3), and pathway enrichment analysis was performed using Metascape (https://metascape.org/gp/index.html). Visualization of selected pathway were generated by R packages ggplot2 (v3.4.3).

## Data availability

The sequencing data reported in this study have been deposited in the NCBI Gene Expression Omnibus (GEO) with the accession number GSE329243, GSE329244, and GSE329245. All other data are available from the corresponding author upon reasonable request.

## Supporting information

This article contains supporting information.

## Conflict of interest

The authors declare that they have no conflicts of interest with the contents of this article.

## References

[1] Bannister A.J., Kouzarides T. (2011). Regulation of chromatin by histone modifications. Cell Res..

[2] Lopez-Hernandez L., Toolan-Kerr P., Bannister A.J., Millan-Zambrano G. (2025). Dynamic histone modification patterns coordinating DNA processes. Mol. Cell.

[3] Agger K., Christensen J., Cloos P.A., Helin K. (2008). The emerging functions of histone demethylases. Curr. Opin. Genet. Dev..

[4] Shi Y., Whetstine J.R. (2007). Dynamic regulation of histone lysine methylation by demethylases. Mol. Cell.

[5] Pedersen M.T., Helin K. (2010). Histone demethylases in development and disease. Trends Cell Biol..

[6] Wang H., Wang L., Erdjument-Bromage H., Vidal M., Tempst P., Jones R.S. (2004). Role of histone H2A ubiquitination in Polycomb silencing. Nature.

[7] de Napoles M., Mermoud J.E., Wakao R., Tang Y.A., Endoh M., Appanah R. (2004). Polycomb group proteins Ring1A/B link ubiquitylation of histone H2A to heritable gene silencing and X inactivation. Dev. Cell.

[8] Cao R., Wang L., Wang H., Xia L., Erdjument-Bromage H., Tempst P. (2002). Role of histone H3 lysine 27 methylation in Polycomb-group silencing. Science.

[9] Pasini D., Bracken A.P., Jensen M.R., Lazzerini Denchi E., Helin K. (2004). Suz12 is essential for mouse development and for EZH2 histone methyltransferase activity. EMBO J..

[10] Margueron R., Justin N., Ohno K., Sharpe M.L., Son J., Drury W.J. (2009). Role of the polycomb protein EED in the propagation of repressive histone marks. Nature.

[11] Swigut T., Wysocka J. (2007). H3K27 demethylases, at long last. Cell.

[12] Agger K., Cloos P.A., Christensen J., Pasini D., Rose S., Rappsilber J. (2007). UTX and JMJD3 are histone H3K27 demethylases involved in HOX gene regulation and development. Nature.

[13] Lan F., Bayliss P.E., Rinn J.L., Whetstine J.R., Wang J.K., Chen S. (2007). A histone H3 lysine 27 demethylase regulates animal posterior development. Nature.

[14] De Santa F., Totaro M.G., Prosperini E., Notarbartolo S., Testa G., Natoli G. (2007). The histone H3 lysine-27 demethylase Jmjd3 links inflammation to inhibition of polycomb-mediated gene silencing. Cell.

[15] Dhar S.S., Lee S.H., Chen K., Zhu G., Oh W., Allton K. (2016). An essential role for UTX in resolution and activation of bivalent promoters. Nucleic Acids Res..

[16] Voigt P., Tee W.W., Reinberg D. (2013). A double take on bivalent promoters. Genes Dev..

[17] Scheuermann J.C., Gutiérrez L., Müller J. (2012). Histone H2A monoubiquitination and Polycomb repression: the missing pieces of the puzzle. Fly.

[18] Sahtoe D.D., van Dijk W.J., Ekkebus R., Ovaa H., Sixma T.K. (2016). BAP1/ASXL1 recruitment and activation for H2A deubiquitination. Nat. Commun..

[19] Wu X., Bekker-Jensen I.H., Christensen J., Rasmussen K.D., Sidoli S., Qi Y. (2015). Tumor suppressor ASXL1 is essential for the activation of INK4B expression in response to oncogene activity and anti-proliferative signals. Cell Res..

[20] Thomas J.F., Valencia-Sanchez M.I., Tamburri S., Gloor S.L., Rustichelli S., Godinez-Lopez V. (2023). Structural basis of histone H2A lysine 119 deubiquitination by Polycomb repressive deubiquitinase BAP1/ASXL1. Sci. Adv..

[21] Gong M., Yuan Y., Dai Z., Lv X., Su J., Huo D. (2024). PRC2 primes bivalent genes for transcription induction independent of histone methyltransferase activity. Sci. China Life Sci..

[22] Nishimura K., Fukagawa T., Takisawa H., Kakimoto T., Kanemaki M. (2009). An auxin-based degron system for the rapid depletion of proteins in nonplant cells. Nat. Methods.

[23] Li R., Huang D., Zhao Y., Yuan Y., Sun X., Dai Z. (2023). PR-DUB safeguards Polycomb repression through H2AK119ub1 restriction. Cell Prolif..

[24] Fursova N.A., Turberfield A.H., Blackledge N.P., Findlater E.L., Lastuvkova A., Huseyin M.K. (2021). BAP1 constrains pervasive H2AK119ub1 to control the transcriptional potential of the genome. Genes Dev..

[25] Conway E., Rossi F., Fernandez-Perez D., Ponzo E., Ferrari K.J., Zanotti M. (2021). BAP1 enhances Polycomb repression by counteracting widespread H2AK119ub1 deposition and chromatin condensation. Mol. Cell.

[26] Bonnet J., Boichenko I., Kalb R., Le Jeune M., Maltseva S., Pieropan M. (2022). PR-DUB preserves Polycomb repression by preventing excessive accumulation of H2Aub1, an antagonist of chromatin compaction. Genes Dev..

[27] Riising E.M., Comet I., Leblanc B., Wu X., Johansen J.V., Helin K. (2014). Gene silencing triggers polycomb repressive complex 2 recruitment to CpG islands genome wide. Mol. Cell.

[28] Yuan W., Wu T., Fu H., Dai C., Wu H., Liu N. (2012). Dense chromatin activates Polycomb repressive complex 2 to regulate H3 lysine 27 methylation. Science.

[29] Blackledge N.P., Rose N.R., Klose R.J. (2015). Targeting Polycomb systems to regulate gene expression: modifications to a complex story. Nat. Rev. Mol. Cell Biol..

[30] Bensaude O. (2011). Inhibiting eukaryotic transcription: which compound to choose? How to evaluate its activity?. Transcription.

[31] Dai Z., Li R., Hou Y., Li Q., Zhao K., Li T. (2021). Inducible CRISPRa screen identifies putative enhancers. J. Genet. Genomics.

[32] Wang S.P., Tang Z., Chen C.W., Shimada M., Koche R.P., Wang L.H. (2017). A UTX-MLL4-p300 transcriptional regulatory network coordinately shapes active enhancer landscapes for eliciting transcription. Mol. Cell.

[33] Herz H.M., Mohan M., Garruss A.S., Liang K., Takahashi Y.H., Mickey K. (2012). Enhancer-associated H3K4 monomethylation by Trithorax-related, the Drosophila homolog of mammalian Mll3/Mll4. Genes Dev..

[34] Kim J.H., Sharma A., Dhar S.S., Lee S.H., Gu B., Chan C.H. (2014). UTX and MLL4 coordinately regulate transcriptional programs for cell proliferation and invasiveness in breast cancer cells. Cancer Res..

[35] Hu D., Gao X., Morgan M.A., Herz H.M., Smith E.R., Shilatifard A. (2013). The MLL3/MLL4 branches of the COMPASS family function as major histone H3K4 monomethylases at enhancers. Mol. Cell Biol..

[36] Zhang Y., Xie G., Lee J.E., Zandian M., Sudarshan D., Estavoyer B. (2024). ASXLs binding to the PHD2/3 fingers of MLL4 provides a mechanism for the recruitment of BAP1 to active enhancers. Nat. Commun..

[37] Biswas S., Lee J.E., Xie G., Masclef L., Ren Z., Cote J. (2025). Colorectal cancer hot spot mutations attenuate the ASXL-MLL4 interaction. J. Biol. Chem..

[38] Wang L., Zhao Z., Ozark P.A., Fantini D., Marshall S.A., Rendleman E.J. (2018). Resetting the epigenetic balance of Polycomb and COMPASS function at enhancers for cancer therapy. Nat. Med..

[39] Lando D., Ma X., Cao Y., Jartseva A., Stevens T.J., Boucher W. (2024). Enhancer-promoter interactions are reconfigured through the formation of long-range multiway hubs as mouse ES cells exit pluripotency. Mol. Cell.

[40] Tosic J., Kim G.J., Pavlovic M., Schroder C.M., Mersiowsky S.L., Barg M. (2019). Eomes and Brachyury control pluripotency exit and germ-layer segregation by changing the chromatin state. Nat. Cell Biol..

[41] Huo D., Yu Z., Li R., Gong M., Sidoli S., Lu X. (2022). CpG island reconfiguration for the establishment and synchronization of polycomb functions upon exit from naive pluripotency. Mol. Cell.

[42] Campagne A., Lee M.K., Zielinski D., Michaud A., Le Corre S., Dingli F. (2019). BAP1 complex promotes transcription by opposing PRC1-mediated H2A ubiquitylation. Nat. Commun..

[43] Scheuermann J.C., de Ayala Alonso A.G., Oktaba K., Ly-Hartig N., McGinty R.K., Fraterman S. (2010). Histone H2A deubiquitinase activity of the Polycomb repressive complex PR-DUB. Nature.

[44] Joo H.Y., Zhai L., Yang C., Nie S., Erdjument-Bromage H., Tempst P. (2007). Regulation of cell cycle progression and gene expression by H2A deubiquitination. Nature.

[45] Nakagawa T., Kajitani T., Togo S., Masuko N., Ohdan H., Hishikawa Y. (2008). Deubiquitylation of histone H2A activates transcriptional initiation via trans-histone cross-talk with H3K4 di- and trimethylation. Genes Dev..

[46] Francois-Campion V., Berger F., Oikawa M., Goumeidane M., Mouniee N., Chenouard V. (2025). Sperm derived H2AK119ub1 is required for embryonic development in Xenopus laevis. Nat. Commun..

[47] Rahman S., Hicks C.W., Gwizdala A., Wolberger C. (2025). Mechanism of USP21 autoinhibition and histone H2AK119 deubiquitination. Sci. Adv..

[48] Yen K., Vinayachandran V., Pugh B.F. (2013). SWR-C and INO80 chromatin remodelers recognize nucleosome-free regions near +1 nucleosomes. Cell.

[49] Huang C., Zhu B. (2014). H3.3 turnover: a mechanism to poise chromatin for transcription, or a response to open chromatin?. Bioessays.

[50] Schlesinger S., Kaffe B., Melcer S., Aguilera J.D., Sivaraman D.M., Kaplan T. (2017). A hyperdynamic H3.3 nucleosome marks promoter regions in pluripotent embryonic stem cells. Nucleic Acids Res..

[51] Chory E.J., Calarco J.P., Hathaway N.A., Bell O., Neel D.S., Crabtree G.R. (2019). Nucleosome turnover regulates histone methylation patterns over the genome. Mol. Cell.

[52] Shpargel K.B., Sengoku T., Yokoyama S., Magnuson T. (2012). UTX and UTY demonstrate histone demethylase-independent function in mouse embryonic development. Plos Genet..

[53] Shpargel K.B., Starmer J., Yee D., Pohlers M., Magnuson T. (2014). KDM6 demethylase independent loss of histone H3 lysine 27 trimethylation during early embryonic development. Plos Genet..

[54] Miller S.A., Mohn S.E., Weinmann A.S. (2010). Jmjd3 and UTX play a demethylase-independent role in chromatin remodeling to regulate T-box family member-dependent gene expression. Mol. Cell.

[55] Vandamme J., Lettier G., Sidoli S., Di Schiavi E., Norregaard Jensen O., Salcini A.E. (2012). The C. elegans H3K27 demethylase UTX-1 is essential for normal development, independent of its enzymatic activity. Plos Genet..

[56] Wang C., Lee J.E., Cho Y.W., Xiao Y., Jin Q., Liu C. (2012). UTX regulates mesoderm differentiation of embryonic stem cells independent of H3K27 demethylase activity. Proc. Natl. Acad. Sci. U. S. A..

[57] Li Q., Wang H.Y., Chepelev I., Zhu Q., Wei G., Zhao K. (2014). Stage-dependent and locus-specific role of histone demethylase Jumonji D3 (JMJD3) in the embryonic stages of lung development. Plos Genet..

[58] Bonnet J., Triantopoulou E., Birnhaupl J., Lu C., Fuller M.T., Muller J. (2025). Histone modification cross-talk and protein complex diversification confer plasticity to Polycomb repression. Genes Dev..

[59] Barradas M., Anderton E., Acosta J.C., Li S., Banito A., Rodriguez-Niedenfuhr M. (2009). Histone demethylase JMJD3 contributes to epigenetic control of INK4a/ARF by oncogenic RAS. Genes Dev..

[60] Nakka K., Hachmer S., Mokhtari Z., Kovac R., Bandukwala H., Bernard C. (2022). JMJD3 activated hyaluronan synthesis drives muscle regeneration in an inflammatory environment. Science.

[61] Seok S., Kim Y.C., Byun S., Choi S., Xiao Z., Iwamori N. (2018). Fasting-induced JMJD3 histone demethylase epigenetically activates mitochondrial fatty acid beta-oxidation. J. Clin. Invest..

[62] Park D.H., Hong S.J., Salinas R.D., Liu S.J., Sun S.W., Sgualdino J. (2014). Activation of neuronal gene expression by the JMJD3 demethylase is required for postnatal and adult brain neurogenesis. Cell Rep..

[63] Wu X., Johansen J.V., Helin K. (2013). Fbxl10/Kdm2b recruits polycomb repressive complex 1 to CpG islands and regulates H2A ubiquitylation. Mol. Cell.

