# Supplementary material for: BAP1 and UTX are dispensable for transcription-driven Polycomb erasure but essential for enhancer establishment
Source: J Biol Chem. 2026 Aug 7;302(10):113415. doi: 10.1016/j.jbc.2026.113415 (PMC13570294; doi:10.1016/j.jbc.2026.113415)
Supplement: Supplementary file 1 [file mmc4.pdf]

FS1

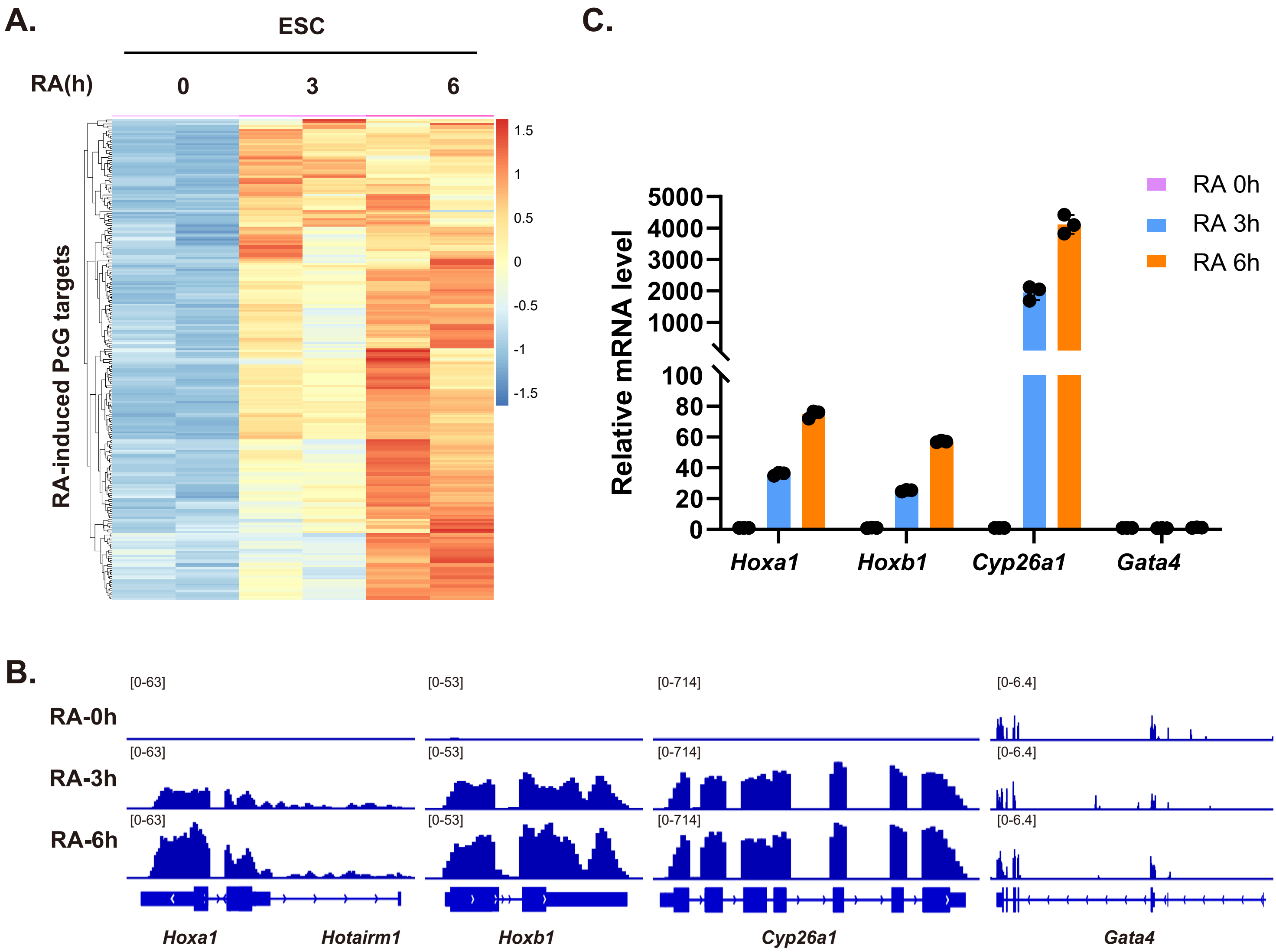

Figure S1. Transcription induction kinetics of RA-inducible PcG target genes in response to retinoic acid.

A. Heatmap showing expression changes of RA-inducible PcG targets upon RA-treated 0, 3, 6 h in WT ESCs.

B. Representative RNA-seq tracks of gene expression before and after RA induction. *Gata4* is served as a negative control.

C. RT-qPCR analysis of *Hoxa1*, *Hoxb1*, *Cyp26a1* mRNA levels at 3 and 6 h of RA treatment in WT ESCs.
