# Supplementary material for: BAP1 and UTX are dispensable for transcription-driven Polycomb erasure but essential for enhancer establishment
Source: J Biol Chem. 2026 Aug 7;302(10):113415. doi: 10.1016/j.jbc.2026.113415 (PMC13570294; doi:10.1016/j.jbc.2026.113415)
Supplement: Supplementary file 2 [file mmc5.pdf]

FS2

A.

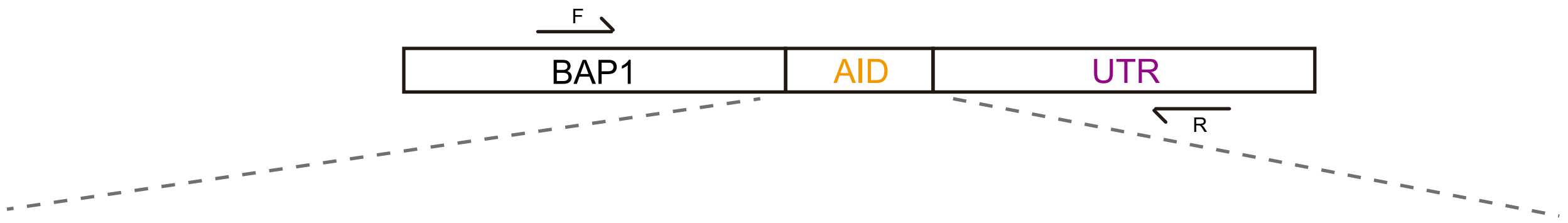

AAGGCCAAACGCCAGATGTACCCATACGACGTCCCAGACTACGCTCCTAAAGATCCAGCCAAACCTCC
GGCCAAGGCACAAGTTGTGGGATGGCCACCGGTGAGATCATACCGGAAGAACGTGATGGTTTCCTGC
CAAAAATCAAGCGGTGGCCCGGAGGCGGCGGCGTTCGTGAAGTGAGAACCTCTGGCCCTGACT

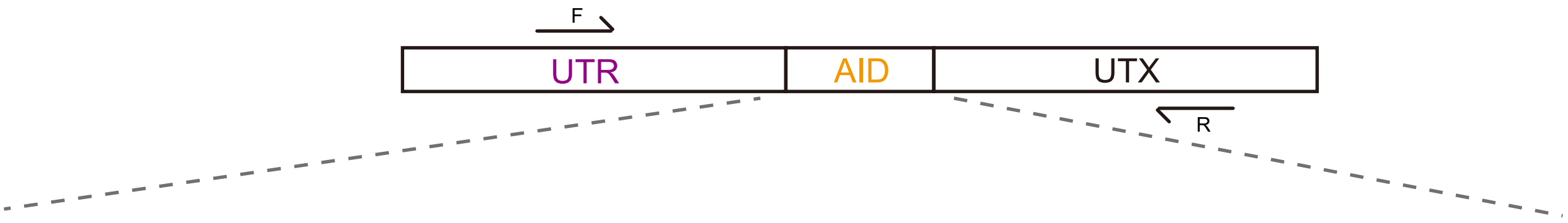

TCGCTGCGTTTCCATGTACCCATACGACGTCCCAGACTACGCTCCTAAAGATCCAGCCAAACCTCCGG
CCAAGGCACAAGTTGTGGGATGGCCACCGGTGAGATCATACCGGAAGAACGTGATGGTTTCCTGCCAA
AAATCAAGCGGTGGCCCGGAGGCGGCGGCGTTCGTGAAGATGAAATCATGCGGAGTGTCGCT

B.

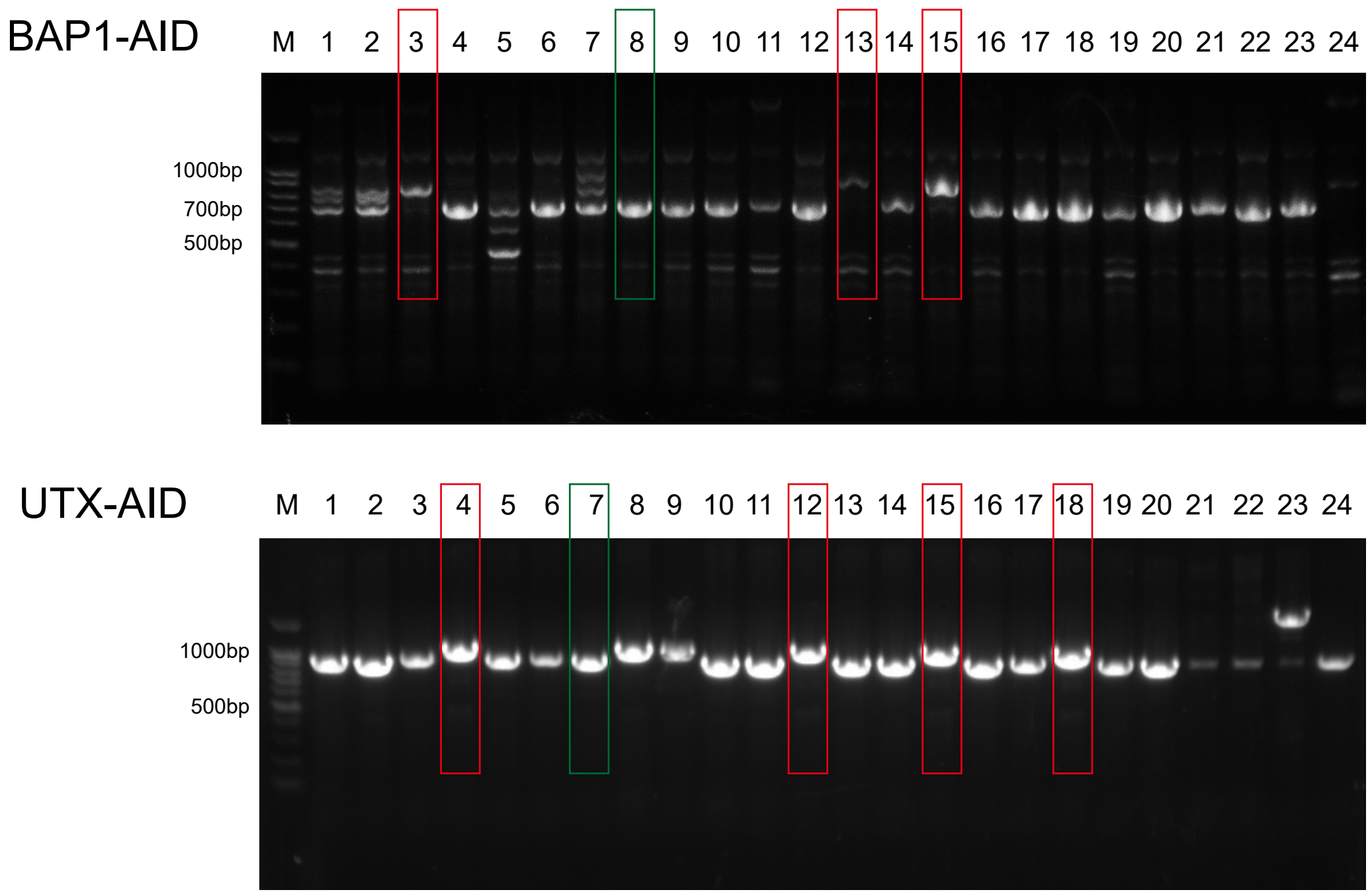

Figure S2. Generation of BAP1-AID and UTX-AID ESCs.

A. Schematic diagram shows the construct strategy of BAP1-AID or AID-UTX ESCs. Endogenous BAP1 or UTX alleles are respectively C or N-terminally fused to the full-length HA-AID tag indicated by orange. The CDS and UTR regions are marked by black and purple respectively. And the termination codon or initiation codon is indicated in light blue. F and R denote genotyping primer pairs.

B. PCR genotyping using primer pairs mentioned in B. DNA marker sizes (bp) are shown on the left. Green boxes indicate WT clones while red boxes indicate positive clones with the HA-AID tag inserted.
