# Supplementary material for: BAP1 and UTX are dispensable for transcription-driven Polycomb erasure but essential for enhancer establishment
Source: J Biol Chem. 2026 Aug 7;302(10):113415. doi: 10.1016/j.jbc.2026.113415 (PMC13570294; doi:10.1016/j.jbc.2026.113415)
Supplement: Supplementary file 3 [file mmc6.pdf]

FS3

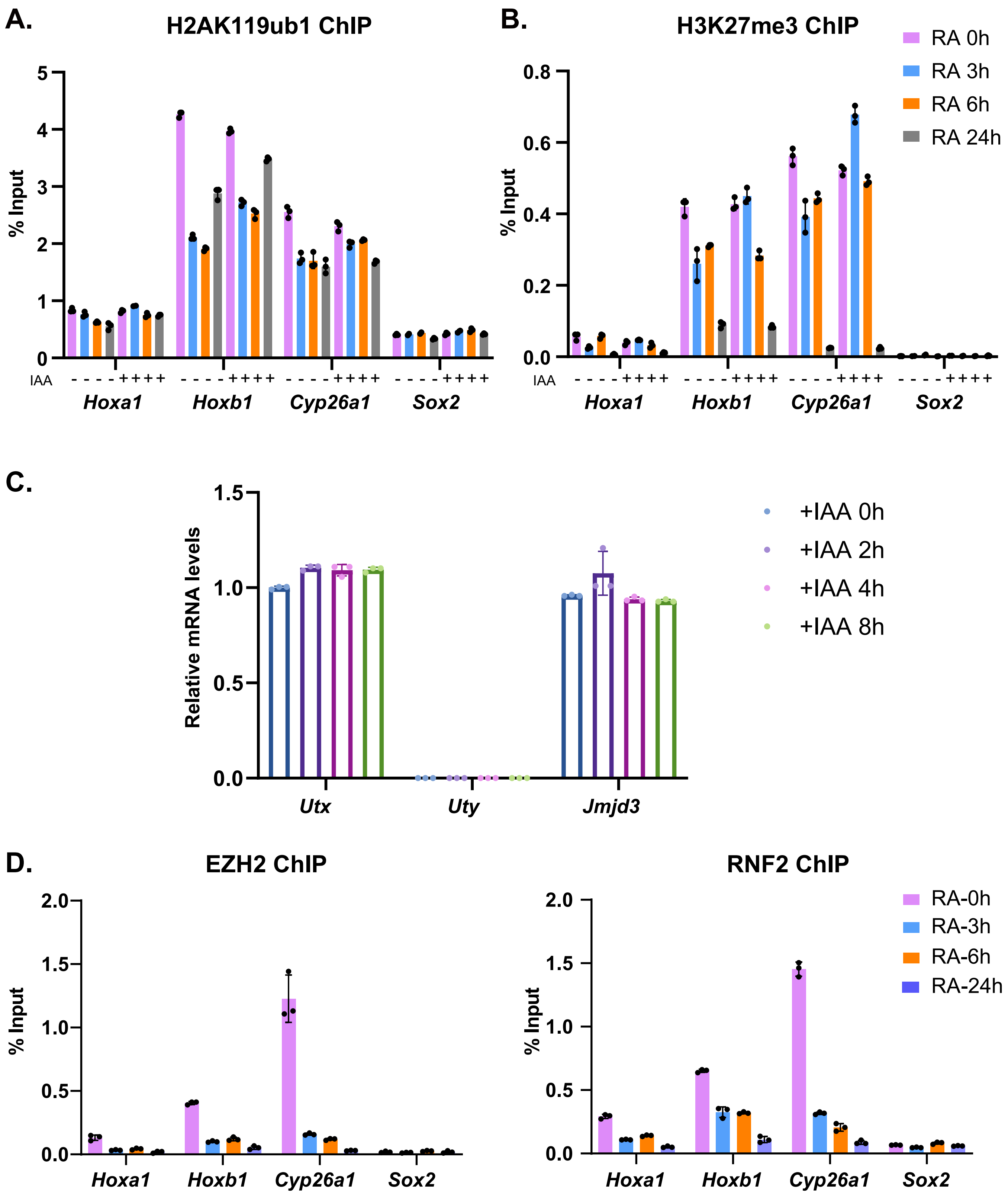

Figure S3. ChIP-qPCR analyses during RA-induced bivalency resolution.
A-B. H2AK119ub1 (A) or H3K27me3 (B) ChIP-qPCR analysis at RA-inducible PcG target gene promoters in BAP1-AID or UTX-AID ESCs treated with or without IAA and stimulated with RA for 0, 3, 6, or 24 h. *Sox2* serves as a negative control.
C. RT-qPCR analysis of *Utx, Uty, Jmjd3* mRNA levels during RA treatment in UTX-AID ESCs.
D. ChIP-qPCR analysis of EZH2 and RNF2 occupancy at promoters of representative bivalent genes following 2 μM RA treatment for 0, 3 or 6 h in WT ESCs.
