# Supplementary material for: BAP1 and UTX are dispensable for transcription-driven Polycomb erasure but essential for enhancer establishment
Source: J Biol Chem. 2026 Aug 7;302(10):113415. doi: 10.1016/j.jbc.2026.113415 (PMC13570294; doi:10.1016/j.jbc.2026.113415)
Supplement: Supplementary file 4 [file mmc7.pdf]

FS4

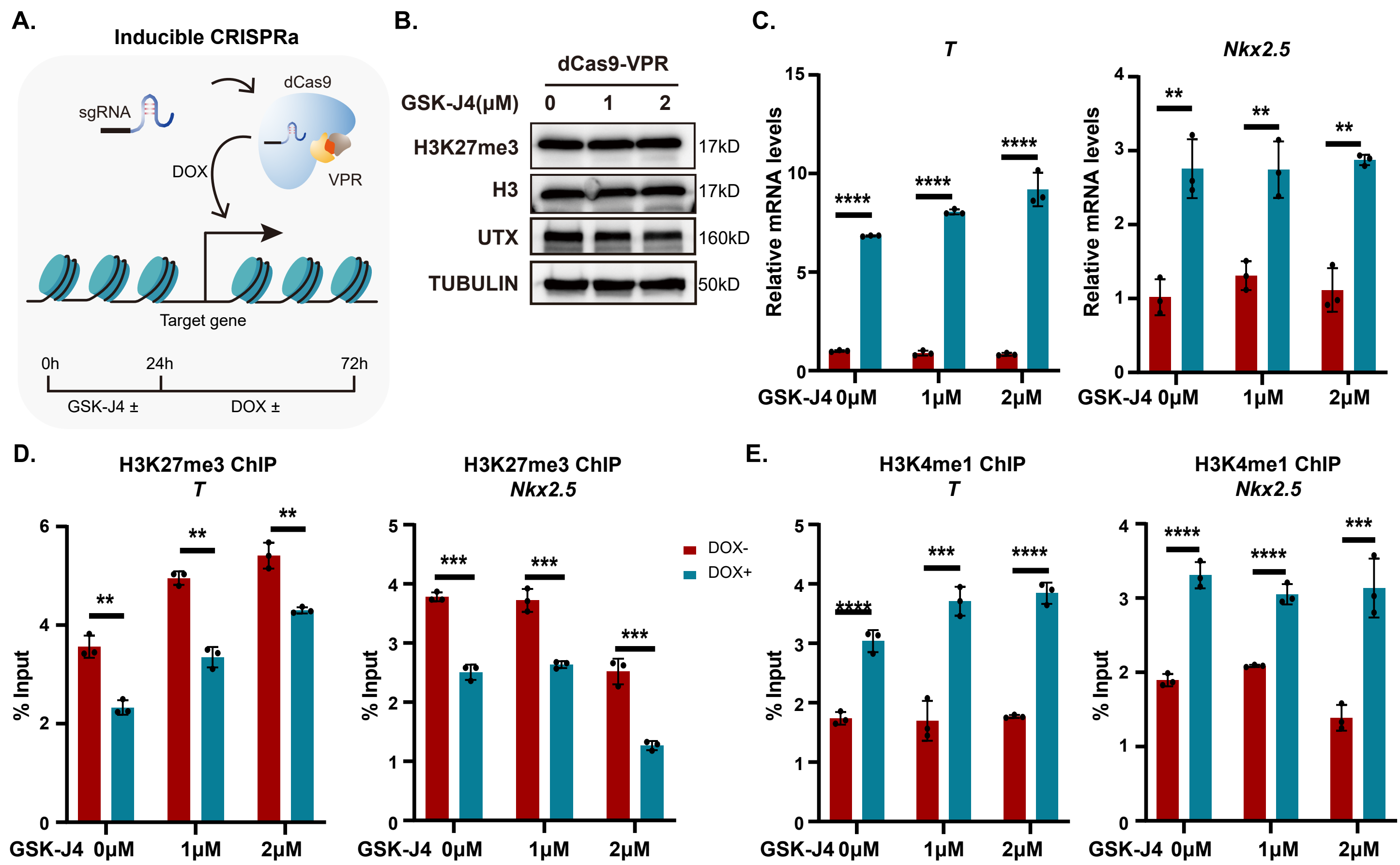

Figure S4. Induced transcription drives loss of H3K27me3 at poised enhancers independent of UTX activity.

A. Schematic diagram shows the strategy of inducible CRISPRa (iVPR) system and the timing of GSK-J4 and Dox treatment.

B. WB assays detecting the UTX and H3K27me3 levels upon GSK-J4 (0, 1, 2 μM) treatment for 24 h.

C. RT-qPCR analysis of destinated genes which sgRNA targeted the enhancer of *T* (left panel) or *Nkx2.5* (right panel) in the iVPR ESCs with the treatment of GSK-J4 and Dox induction for 48 h. The error bars denote SD; n = 3. P value was calculated by two-sided unpaired t test. *, P < 0.05, **, P < 0.01; ***, P < 0.001.

D-E. ChIP-qPCR analysis of H3K27me3 or H3K4me1 enrichment at the enhancer regions of *T* (E) and *Nkx2.5* (F) after GSK4-J4 treatment and DOX induction for 48 h in iVPR ESCs. The error bars denote SD; n = 3. P value was calculated by two-sided unpaired t test. *, P < 0.05, **, P < 0.01; ***, P < 0.001.
