# Supplementary material for: BAP1 and UTX are dispensable for transcription-driven Polycomb erasure but essential for enhancer establishment
Source: J Biol Chem. 2026 Aug 7;302(10):113415. doi: 10.1016/j.jbc.2026.113415 (PMC13570294; doi:10.1016/j.jbc.2026.113415)
Supplement: Supplementary file 5 [file mmc8.pdf]

FS5

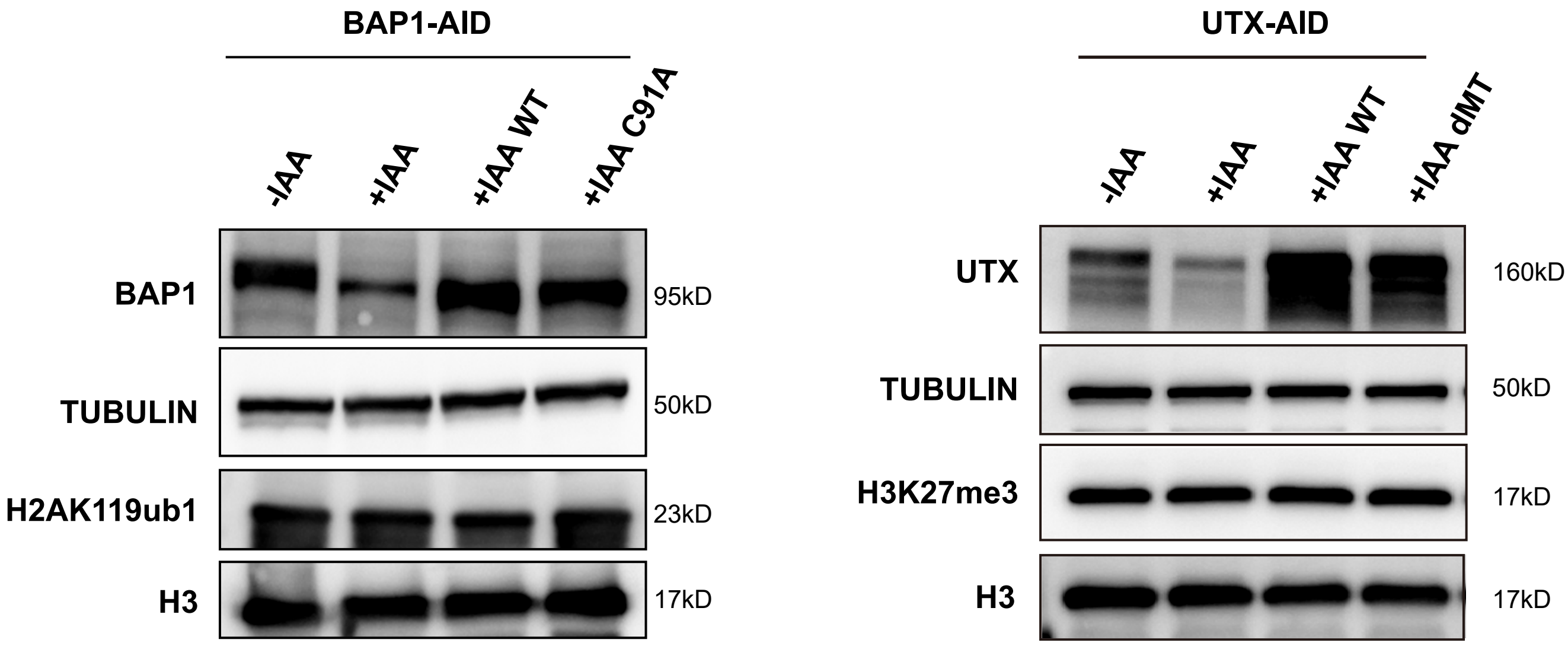

Figure S5. Characterization of the rescue systems.
WB analysis of indicated proteins in BAP1-AID or UTX-AID ESCs reconstituted with WT or catalytically inactive mutant of BAP1 or UTX, with IAA treatment. Tubulin and H3 serve as the loading control.
