# Supplementary material for: BAP1 and UTX are dispensable for transcription-driven Polycomb erasure but essential for enhancer establishment
Source: J Biol Chem. 2026 Aug 7;302(10):113415. doi: 10.1016/j.jbc.2026.113415 (PMC13570294; doi:10.1016/j.jbc.2026.113415)
Supplement: Supplementary file 6 [file mmc9.pdf]

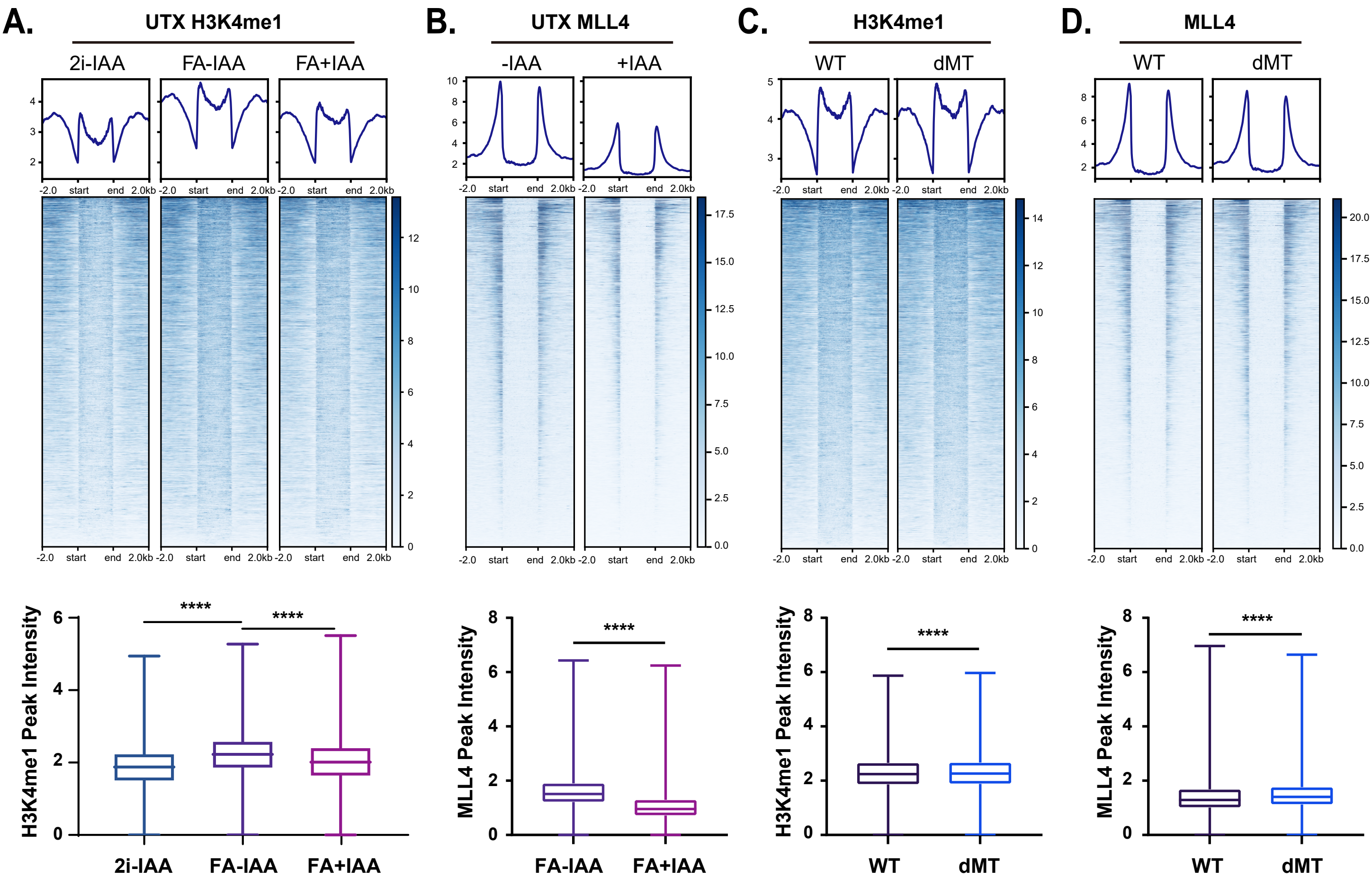

Figure S6. UTX regulates enhancer establishment independent of its demethylase activity.

A. Heatmaps of H3K4me1 ChIP-seq signals at H3K4me1-gain peaks in control (-IAA) and UTX-depleted (+IAA) AID ESCs under 2i and 24 h FA conditions. The lower panel presents the associated statistics. Data are presented as boxplots; error bars represent standard deviation (SD). Statistical significance was determined by Wilcoxon rank-sum test: * $P < 0.05$, ** $P < 0.01$, *** $P < 0.001$, **** $P < 0.0001$. This statistical method was applied to all subsequent panel.

B. Heatmaps showing MLL4 CUT&Tag signals at H3K4me1-gain regions during 24 h FA-induced differentiation UTX-AID ESCs with IAA treatment. Statistical analysis for the above data is provided in the lower panel.

C-D. Heatmaps showing H3K4me1 ChIP-seq or MLL4 CUT&Tag signals in reintroduced WT and dMT(H1146A E1148A) UTX-AID ESCs with IAA treatment during 24 h FA differentiation. The corresponding statistical data are displayed in the lower panel.
