# Supplementary material for: BAP1 and UTX are dispensable for transcription-driven Polycomb erasure but essential for enhancer establishment
Source: J Biol Chem. 2026 Aug 7;302(10):113415. doi: 10.1016/j.jbc.2026.113415 (PMC13570294; doi:10.1016/j.jbc.2026.113415)
Supplement: Supplementary file 7 [file mmc3.docx]

**Supporting Information**

**BAP1 and UTX are dispensable for transcription-driven Polycomb erasure but essential for enhancer establishment**

Meihan Gong^1,3,4^, Xuanyuan Li^1,2,4^, Shaomei Chen^1,2^, Xudong Wu^1,2,5,*^

^1^Tianjin Medical University Cancer Institute & Hospital, National Clinical Research Center for Cancer, Tianjin’s Clinical Research Center for Cancer, The Province and Ministry Co-sponsored Collaborative Innovation Center for Medical Epigenetics, State Key Laboratory of Druggability Evaluation and Systematic Translational Medicine, Key Laboratory of Cancer Prevention and Therapy, Tianjin 300060, China.

^2^Department of Cell Biology, Tianjin Medical University, Qixiangtai Road 22, Tianjin 300070, China.

^3^State Key Laboratory of Experimental Hematology, Key Laboratory of Immune Microenvironment and Disease (Ministry of Education), Tianjin Key Laboratory of Medical Epigenetics, Tianjin Medical University Cancer Institute and Hospital, Tianjin Medical University, Tianjin 300070, China.

^4^These authors contributed equally.

^5^Lead contact

*For correspondence: wuxudong@tmu.edu.cn (X.W.)

**Supplementary figures**

**Table S1. RT-qPCR and ChIP-qPCR primers used in this study**

**Table S2. Reagents used in this study**
